# VALIDITY OF THE BRAZILIAN-PORTUGUESE VERSION OF MOOREHEAD-ARDELT QUALITY OF LIFE QUESTIONNAIRE II AMONG PATIENTS WITH SEVERE OBESITY

**DOI:** 10.1590/0102-672020230049e1767

**Published:** 2023-10-13

**Authors:** Mariane de Carvalho Cremonesi, Leorides Duarte-Guerra, Denis Pajecki, Marco Aurelio Santo, Francisco Lotufo, Yuan-Pang Wang

**Affiliations:** 1 Universidade de São Paulo, Department of Psychiatry – São Paulo (SP), Brazil; 2 Universidade de São Paulo, Department of Gastroenterology – São Paulo (SP), Brazil

**Keywords:** Bariatric surgery, Quality of life, Obesity, morbid, Validation study, Cirurgia bariátrica, Qualidade de vida, Obesidade mórbida, Estudo de validação

## Abstract

**BACKGROUND:**

Patients with obesity present multiple comorbid psychiatric conditions and experience impairments in health-related quality of life. Reliable and valid tools that evaluate health-related quality of life are essential for clinical practice.

**AIMS:**

This study aimed to investigate the reliability and validity of the six-item Moorehead-Ardelt Quality of Life Questionnaire II among Brazilian patients with severe obesity.

**METHODS:**

We assessed 387 patients (mean age 43 years, 78.8% women, mean body mass index of 46.5 kg/m²) on the waiting list of a bariatric surgery center. Trained research assistants concurrently applied the Moorehead-Ardelt Quality of Life-II, the Montgomery-Åsberg Depression Rating Scale, and the Global Assessment of Functioning for assessing health-related quality of life, comorbid depressive symptoms, and patient functioning level, respectively.

**RESULTS:**

The internal consistency of the Moorehead-Ardelt Quality of Life-II was considered acceptable. The total score was correlated with the severity of depressive symptoms and functioning level. The more body mass index increases, the more health-related quality of life worsens. The Moorehead-Ardelt Quality of Life-II presented a unidimensional structure.

**CONCLUSIONS:**

The unidimensional Moorehead-Ardelt Quality of Life-II is a reliable and valid measure for evaluating health-related quality of life in Brazilian patients with severe obesity. The questionnaire allows to quickly assess the health-related quality of life of patients in different bariatric contexts, considering depression and functional level.

## RESUMO

**RACIONAL:**

Pacientes com obesidade apresentam múltiplas condições psiquiátricas comórbidas e experienciam prejuízos na qualidade de vida relacionada à saúde. Ferramentas confiáveis e válidas que avaliam a qualidade de vida relacionada à saúde são essenciais para a prática clínica.

**OBJETIVOS:**

Este estudo teve como objetivo investigar a confiabilidade e validade do Moorehead-Ardelt Quality of Life-II de seis itens entre pacientes com obesidade grave.

**MÉTODOS:**

Foram avaliados 387 pacientes (idade média de 43 anos, 78,8% mulheres, índice de massa corporal (IMC) médio de 46,5 kg/m², na lista de espera de um centro cirurgia bariátrica. Assistentes de pesquisa treinados aplicaram simultaneamente o Moorehead-Ardelt Quality of Life-II, a Escala de Depressão de Montgomery-Åsberg e a Avaliação Global do Funcionamento para avaliar, respectivamente, a qualidade de vida relacionada à saúde, os sintomas depressivos comórbidos e o nível funcional do paciente.

**RESULTADOS:**

A consistência interna do Moorehead-Ardelt Quality of Life-II foi considerada aceitável. A pontuação total do Moorehead-Ardelt Quality of Life-II foi correlacionada com a gravidade dos sintomas depressivos e nível funcional. Quanto maior o IMC, menor a qualidade de vida relacionada à saúde. O Moorehead-Ardelt Quality of Life-II apresentou uma estrutura unidimensional.

**CONCLUSÕES:**

O questionário Moorehead-Ardelt Quality of Life-II unidimensional é confiável e válido na avaliação da qualidade de vida relacionada à saúde em pacientes brasileiros com obesidade grave. O questionário permite avaliar rapidamente a qualidade de vida relacionada à saúde dos pacientes em diferentes contextos, considerando depressão e nível funcional.

## INTRODUCTION

Obesity is a major global health challenge^24^ and its burden negatively influences disability-adjusted life-years, years of life lost, and additional millions of deaths worldwide. The low health-related quality of life (HRQoL) is one of the reasons why people who live with dysfunctional obesity seek bariatric surgery^17^. Therefore, reliable and validated instruments are crucial to evaluate the HRQoL of obese patients, at all stages of bariatric surgery. In middle-upper income countries like Brazil, 22.4% of adults already present a mean body mass index (BMI) of 30 kg/m2^4^. Although the demand for bariatric surgery is rapidly growing, access to bariatric surgery is restricted due to extensive search for treatment^3^. It is forecast that low- and middle-income countries will soon reach a similar level of obesity, as high as reported for a high-income country like the United States, where the prevalence of obesity already reaches 36.2% of the population^31^.

There are several definitions of HRQoL^16^. The World Health Organization (WHO) has one of the most comprehensive definitions of quality of life (QoL), which encompasses several functional domains. The self-perception of one's social context, goals, expectations, and concerns are concepts related to the QoL that affect the physical, psychological, and social domains of health^29^. These specific domains have been widely defined as “health-related quality of life”^16^. Although there is no consensus on its concept and which domains it encompasses^16^, the interference of mental health in daily role functioning is undeniable. Frequently, psychiatric symptoms exert a disabling influence on the patient's general functioning^13,23^. It could be expected that patients with comorbidities, severe obesity, and psychiatric disorders would present even worse HRQoL^14^.

Commonly associated comorbidities, such as psychiatric disorders and obesity, can create a negative spiral effect on HRQoL^17^. This association seems to be bidirectional and its joint effect on the patient's functionality is of great concern. Previous studies have shown that people with greater degrees of obesity experience significant impairments when associated with mental disorders^9,17^. Contrariwise, losing weight has been shown to improve the QoL of obese people, regardless of the type of treatment. Patients with severe obesity present a high frequency of psychiatric disorders throughout life (up to 80%)^9,11^. Mood disorders are among the most frequent conditions, estimated at 23%^11^.

Few studies evaluated the association between psychiatric disorders and QoL in patients with severe obesity. Among existing tools, the Short Form 36 Health Survey (SF-36) and the Rand's 36-Item Health Survey (RAND-36) are common questionnaires for assessing HRQoL^20^. However, these tools were not developed for the purpose of assessing obese patients. The Moorehead-Ardelt Quality of Life Questionnaire II (MA-II)^22,25^ is a popular instrument accepted for evaluating bariatric surgery outcomes. This brief tool has a good convergent validity with SF-36 and Impact of Weight Quality of Life-Lite (IWQoL-Lite)^22^. Although the MA-II has been widely used among Portuguese-speaking respondents^5,19^, there is no formal demonstration of its reliability and validity.

An assessment tool of HRQoL that is sensitive to aspects of the patient's mental health is fundamental to provide health professionals with an accurate measurement to monitor HRQoL in patients with severe obesity.

The present study aimed to validate the MA-II for use among Brazilian-Portuguese-speaking patients with severe obesity seeking bariatric surgery. The reliability and convergent validity of MA-II were evaluated with concurrent measures of depressive symptoms and global functioning as surrogates of HRQoL. We also explored the dimensionality of the constructs covered by this tool. Potential factors affecting MA-II scores were estimated through regression models.

## METHODS

### Study design and sampling

This is a validation study to evaluate the psychometric properties of an HRQoL tool among patients with severe obesity. Research assistants recruited 500 consecutive patients from the waiting list of a university-based bariatric center. Inclusion criteria were fluency in Brazilian-Portuguese and severe obesity (BMI>40). Exclusion criteria were evidence of psychosis or intellectual disability, age below 18 years, illiterate people, or those unable to attend the bariatric center due to mobility or geographical issues. During telephone contact, 63 individuals declined to participate. Additional 44 exclusions were done owing to mobility difficulty (n=37), severe psychiatric illness (n=2), or previous bariatric surgery (n=5). In total, 393 eligible patients agreed to participate in the study (75.6%), but only 378 returned the complete form. All data were cross-sectionally recorded in person during a scheduled encounter in the ambulatory.

The Institutional Ethics Committee approved the study (#0228/11). All participants signed informed consent before entering the study.

### Assessment tools

The MA-II^22,25^ assesses outcomes of bariatric surgery through different indicators of HRQoL. This self-report questionnaire evaluates six domains of HRQoL: (Q1) self-esteem; (Q2) physical exercise; (Q3) social contact; (Q4) affective relationships; (Q5) job performance; and (Q6) relationship with food. Respondents are asked to mark an answer sheet with simple visual cues after a brief statement. The ten-item Likert score ranges from −5 to +5, with higher scores indicating better HRQoL. The total score of the six-item MA-II categorizes respondents’ HRQoL as very poor (−3 to −2.1), poor (−2 to −1.1), fair (−1 to +1), good (+1.1 to +2), or very good (+2.1 to +3). The questionnaire takes between two and five minutes to complete. We obtained authorization for use from the copyright holder of the instrument.

The original English version of MA-II was subjected to translation by three bilingual professionals. A native English translator back-translated the MA-II. During the scrutiny of figures and symbols of the answer form, Q3 yielded an unclear statement “I have satisfactory social contacts”, which could be understood either as satisfaction with the quality or number of social contacts. Further concern refers to the suitable use of overlapping emojis (e.g., Q1 “usually I feel”, Q2 “physical activities”, and Q5 “pleasure in sexual relations”), which could not be readily understood by all respondents from different age brackets and educational levels. However, no participant reported comprehension problems throughout the pilot application and cognitive debriefing. During a panel discussion, we decided to translate Q3 as “I have satisfactory social contacts” (“*tenho contatos sociais satisfatórios*”) without further adaptation of emojis.

The Montgomery-Åsberg Depression Rating Scale (MADRS)^10,21,30^ rates the presence of symptoms of depressive disorder through an anchored clinical interview^30^. This ten-item tool ascertains the biological, cognitive, affective, and behavioral aspects of depression. The total score classifies patients in the following levels of severity: normal or absent 0–6; mild 7–19; moderate 20–34; and severe 35–60. The MADRS scale takes between 10 and 15 minutes to complete. The version we used was validated for the Brazilian-Portuguese-speaking population^10^ and obese participants^12^.

The Global Assessment of Functioning (GAF) scale^1,2,26^ evaluates the patient's global functioning, as a measure of the severity of psychiatric disorders^8,13,26^. The GAF assesses whether the presence of psychiatric disorders would interfere with the instrumental functioning of patient's daily life. The GAF score ranges from 1 to 100, and the rater should endorse a specific range of functioning with the help of descriptive cues in the form. A low rating indicates poor functioning, whereas a high rating indicates good functioning. The GAF is a reliable assessment tool with good sensitivity and specificity^1^ and is associated with HRQoL^1,23,26^.

### Statistical analysis

Firstly, the descriptive analyses of MA-II, GAF, and MADRS were performed. Data were presented as mean (M), standard deviation (SD), and frequency (%).

In addition, we calculated the MA-II scores and categorized the totals as very poor, poor, fair, good, or very good. A chi-square (χ²) analysis was performed to evaluate differences between MA-II categories and gender classifications. The analysis of the MA-II items was performed through Spearman's rank correlation between MA-II items and scales of psychopathology (MADRS) and functioning (GAF) scores.

Initially, a simple linear regression model was used to evaluate if the HRQoL score could predict MADRS and GAF scores. However, the residual analysis showed an asymmetric distribution of data. Therefore, the extension of generalized additive models for location, scale, and shape (GAMLSS) was used to adjust the models. The GAMLSS are univariate distributional regression models, where all parameters of the assumed distribution for the response can be modeled as additive functions of the explanatory variables. The zero-adjusted gamma (ZAGA) distribution was fitted for MADRS and the skew-normal type 2 (SN2) distribution for GAF in GAMLSS models. The skew-exponential power type 4 (SEP4) distribution was fitted for the dependent variable BMI. The following parameters determined the model fitness, namely scaling parameter estimation (o); asymmetry parameter estimation (?), and the Akaike information criterion (AIC). A stepwise strategy was applied to select significant characteristics associated with the MA-II total score.

A confirmatory factor analysis (CFA) was performed, assuming a unidimensional structure for the MA-II. The maximum likelihood estimation (MLM) method with robust pattern errors estimated the dimensional model. The Satorra-Bentler adjustment was applied to correct statistical errors. The model's goodness-of-fit was assessed through the comparative fit index (CFI) and Tucker-Lewis index (TLI), with values greater than 0.95 indicating good fitness. Additionally, root mean square error of approximation (RMSEA) values less than 0.06 indicated satisfactory model fitness. The CFA was fitted using the *cfa* function of the *lavaan* package of R software. The alternative McDonald's omega of reliability was estimated after the factorial model to correct the underestimation bias of α, due to violation of the assumption of tau-equivalence and covariance error.

The data analysis was conducted with Statistical Package for Social Sciences (SPSS) version 18.0 (https://www.ibm.com/br-pt/products/spss-statistics) and with software R 4.0.2 (www.r-project.org). The significance level adopted in all analyses was 5% for two-tailed tests.

## RESULTS

Of the 378 patients in the final sample who completed the MA-II questionnaire, most were women (78.8%) and married (51.3%). The mean age of the participants was 43.0 years (SD=11.5), and the median BMI was 46.5 kg/m² (range: 31.2 to 92.1). Men presented a higher BMI than women (50.2 *vs*. 46.4; p=0.01) (Table 1).

**Table 1:** Socio-demographic and clinical variables of participants (n=378) on the waiting list.

Variable		n	(%)
Women		298	78.8
Men		80	21.2
Age			
	Mean (SD)	43	(11.5)
	Median (min-max)	43	(18–73)
Marital status			
	Married	194	51.3
	Widower	24	6.3
	Divorced	62	16.4
	Not married	98	25.9
Scholarity			
	Elementary school	135	35.7
	High school	169	44.7
	Graduate	67	17.7
	Postgraduate	7	1.8
Employed (yes)		243	64.2
BMI			
	Mean (SD)	47.2 (7.4)	
	Median (min; max)	46.5 (31.2–92.1)	

BMI: body mass index; SD: standard deviation.

The median total score of the MA-II was 0.9 (range: −2.9 to 3), the MADRS 2.0 (range: 0 to 58), and the GAF 80 (range: 40 to 98). For interpretation, respondents reported a fair HRQoL as measured by MA-II, good global functioning by GAF, and mild level of depressive symptoms by MADRS (Table 2).

**Table 2:** Distribution and dispersion of scores of Moorehead-Ardelt Quality of Life Questionnaire II, Global Assessment Functioning, and Montgomery-Åsberg Depression Rating Scale scales.

Variable		
MA-II		
	Mean (SD)	0.8 (1.2)
	Median (min; max)	0.9 (−2.9; 3)
GAF		
	Mean (SD)	76.8 (12.3)
	Median (min; max)	80 (40; 98)
MADRS		
	Mean (SD)	7.7 (11.3)
	Median (min; max)	2 (0; 58)

MA-II: Moorehead-Ardelt Quality of Life Questionnaire II; GAF: Global Assessment Functioning; SD: standard deviation; MADRS: Montgomery-Åsberg Depression Rating Scale; Min: minimum; max: maximum.

Table 3 displays the categorization of levels of self-reported QoL according to the total score of the MA-II. For the total sample, around half of the patients (49.2%) reported fair QoL, while 43.2% had a good or very good QoL, and 7.7%, poor or very poor. A similar pattern of QoL was observed in both women and men sub-group (p=0.30). Regarding internal consistency, the estimated McDonald's Omega was 0.62.

**Table 3:** Frequency of categories of quality-of-life rating, according to the total score of the MA-II scale.

	Total n=378 (%)	Women n=299 (%)	Men n=88 (%)*
Very good	55 (14.6)	41 (13.7)	15 (17.0)
Good	108 (28.6)	84 (28.1)	27 (30.7)
Fair	186 (49.2)	147 (49.2)	44 (50.0)
Poor	18 (4.8)	17 (5.7)	1 (1.1)
Very poor	11 (2.9)	10 (3.3)	1 (1.1)

* Chi-square: χ² (df=4) = 4.882; p=0.30.

Table 4 indicates that the correlation between most MA-II items was significant (p<0.01), except for the item job performance (Q4) with physical activities (Q2) and eating behavior (Q6). Although each item of the Brazilian-Portuguese MA-II was significantly correlated with all other items in this scale, we observed a salient ceiling effect of 1.58% (six patients).

**Table 4:** Correlation matrix between the items of the MA-II questionnaire for 378 patients with obesity on the waiting list for bariatric surgery

	Q1	Q2	Q3	Q4	Q5	Q6
Q1: Self-esteem	1.000	0.179*	0.367*	0.168*	0.363*	0.292*
Q2: Physical exercise		1.000	0.319*	0.053	0.188*	0.287*
Q3: Social contact			1.000	0.279*	0.417*	0.301*
Q4: Job performance				1.000	0.348*	0.099
Q5: Affective relationships					1.000	0.190*
Q6: Relationship with food						1.000

Spearman's correlation: *p<0.01; Q: questions.

Table 5 shows the correlation between all six MA-II items with the construct of depression (MADRS) and global functioning (GAF). All domains of MA-II were correlated with MADRS and GAF (p<0.001), suggesting a sound convergent validity. Furthermore, the BMI was correlated with the item Q2 physical activity (p<0.001) and Q4 job performance (p<0.05). These results indicated that high BMI correlated with the patient's satisfaction with physical exercise and capacity to work.

**Table 5:** Correlation between items of the Moorehead-Ardelt Quality of Life Questionnaire II with Montgomery-Åsberg Depression Rating Scale, Global Assessment Functioning, and body mass index for patients with obesity on the waiting list for bariatric surgery (n=378).

MA-II Item	MADRS	GAF	BMI
Q1: Self-esteem	-0.332*	0.290*	0.060
Q2: Physical activity	-0.165*	0.154*	-0.166*
Q3: Social contact	-0.326*	0.271*	0.012
Q4: Job performance	-0.146*	0.180*	-0.103†
Q5: Affective relationships	-0.265*	0.133*	-0.032
Q6: Relationship with food	-0.144*	0.179*	-0.045
Total	-0.368*	0.331*	-0.088

* p<0.01;

† p<0.05;

MA-II: Moorehead-Ardelt Quality of Life Questionnaire II; MADRS: Montgomery-Åsberg Depression Rating Scale; GAF: Global Assessment Functioning; BMI: body mass index; p: p-value.

Table 6 presents results of the traditional linear regression for MADRS, GAF, and BMI. Thereafter, findings of GAMLSS regression models (Table 7) were compared with linear models. GAMLSS models have shown slightly better results, respectively ß=-2.991 (standard error [SE]: 0.540) for MADRS; ß=2.578 (SE: 0.487) for GAF; and ß=-0.016 (SE: 0.008) for BMI. Overall adjustment indicators (o, ѵ, t, and AIC) favored GAMLSS models. The adjustment of non-normal or skewed data distribution, namely ZAGA, SN2, and SEP4, also supported the plausibility of our final models. The ß regression coefficient indicated a negative association between the response (MADRS) and the predictor variable (MA-II), i.e., as the MADRS score increases, the MA-II score decreases. The ß regression coefficient indicated a positive association between the response of GAF and the predictor variable (MA-II), i.e., as the GAF score increases, the MA-II score also increases. The BMI was negatively associated with MA-II.

**Table 6:** Simple linear regression model between the scores of the Moorehead-Ardelt Quality of Life Questionnaire II and the scores of Montgomery-Åsberg Depression Rating Scale, Global Assessment Functioning, and body mass index among patients with obesity on the waiting list (n=378).

	MADRS	GAF	BMI
	β (SE)	β (SE)	β (SE)
Intercept	10.808 (0.637)	74.478 (0.689)	1.833 (0.398)
MA-II	-3.935 (0.446)	3.625 (0.482)	-0.024 (0.008)
	10.331 (1.038)	11.175 (1.038)	1.196 (1.037)
AIC	2,769.006	2,826.832	1,215.721

MADRS: Montgomery-Åsberg Depression Rating Scale; GAF: Global Assessment Functioning; BMI: body mass index; SE: standard error; MA-II: Moorehead-Ardelt Quality of Life Questionnaire II; AIC: Akaike information criterion.

**Table 7:** Generalized additive models for location, scale, and shape between the score of the Moorhead-Ardelt Quality of Life, version II and the score of Montgomery-Åsberg Depression Rating Scale, Assessment of Global Functioning, and body mass index among obese patients on the waiting list (n=378).

		MADRS*	GAF†	BMI‡
		ß (SE)	ß (SE)	ß (SE)
Intercept		14.848 (0.975)	84.045 (1.766)	1.636 (0.372)
MA-II		-2.991 (0.540)	2.578 (0.487)	-0.016 (0.008)
	o	0.861 (1.045)	9.156 (1.078)	1.720 (1.065)
	ѵ	0.427 (0.526)	0.565 (1.126)	1.612 (1.119)
	t			3.910 (1.238)
AIC		1,999.385	2,790.675	1,195.874

MADRS: Montgomery-Åsberg Depression Rating Scale; GAF: Assessment of Global Functioning; BMI: body mass index; SE: standardized error; MA-II: Moorhead-Ardelt Quality of Life, version II; o: scaling parameter estimation; ѵ and t: asymmetry parameter estimation; AIC: Akaike Information Criterion; GAMLSS: Generalized additive models for location, scale, and shape.

* ZAGA: Zero Adjusted Gamma distribution or left-skewed model of GAMLSS;

† SN2: Skew Normal type 2 distribution or right-skewed model of GAMLSS;

‡ SEP4: Skew Exponential Power type 4 or left-skewed model of GAMLSS. The sex of the respondent was included as a covariate, but not significantly associated with BMI.

The CFA model that assumes a unidimensional structure for the MA-II presented CFI and TLI below values considered satisfactory (?²=38.6, CFI=0.887, TLI=0.811, and RMSEA=0.093). However, the model adjustments provided a CFI value of around 0.90, which may be considered a reasonable fitness. These indicators marginally demonstrate a unidimensional structure for the MA-II.

**Figure 1:**
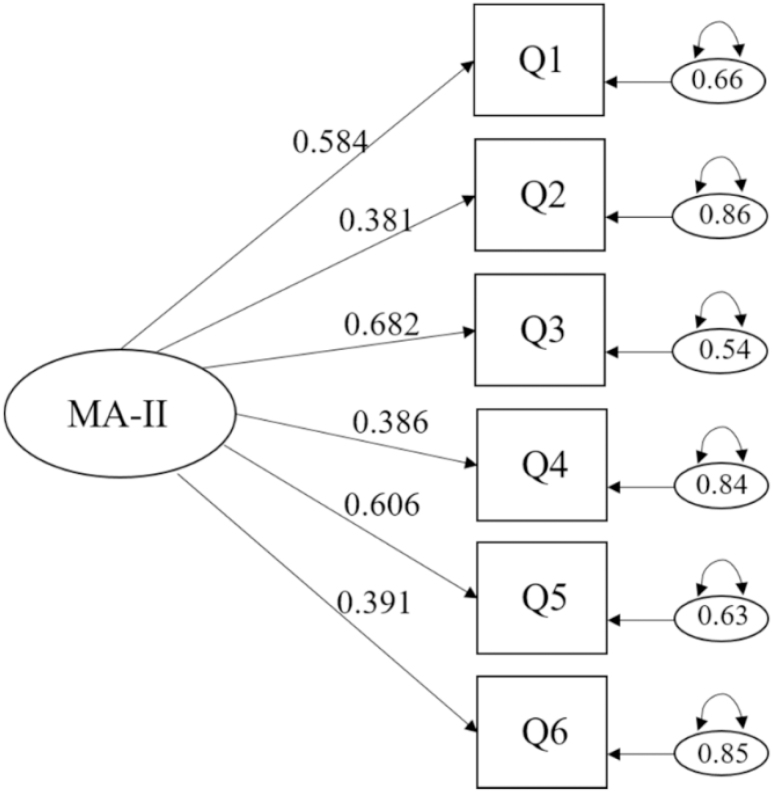
Factor structure of the Moorehead-Ardelt Quality of Life Questionnaire II for the one-dimensional theoretical model. MA-II = Moorehead-Ardelt Quality of Life Questionnaire II; Q: question.

Figure 1 is the path diagram and shows the factor loadings of the MA-II questionnaire. All the items presented standardized factor loadings above 0.3 (?: 0.38–0.68). Items Q1, Q3, and Q5 loaded above 0.5 and the remaining items Q2, Q4, and Q6 loaded around 0.4.

## DISCUSSION

To the best of our knowledge, this is the first validation study of the Brazilian-Portuguese version of MA-II among patients with severe obesity. Although several articles reported the utility of applying MA-II, none investigated the psychometric properties of this tool in Brazilian-Portuguese-speaking patients. While most studies reported only the MA-II score, our study advanced toward its reliability, convergent and factorial validity.

In our study, 92.4% of patients reliably reported fair to very good HRQoL and only 7.6% reported poor or very poor HRQoL. The unidimensional construct covered by the MA-II was associated with clinician-rated depressive symptoms and global functioning. The magnitude of BMI was also associated with poor HRQoL, mainly in the domains of physical exercise (Q2) and job performance (Q4). The MA-II is a brief questionnaire and the present study suggested its cost-effective applicability across all stages of the bariatric procedure. Bearing in mind its convergent validity with the constructs of depression and global functioning, its routine application could contribute to monitoring HRQoL in many obese patients.

Globally, MA-II is an easy HRQoL tool to understand and apply^22^. In most non-English adaptations, the internal consistency of the questionnaire was good or satisfactory^6,7,18,19,27,28^, ranging from 0.72 to 0.88. The internal consistency analysis of MA-II found in this study was 0.62. However, we evaluated the internal consistency using the alternative McDonald's omega coefficient rather than the traditional Cronbach's alpha. The omega relies on fewer assumptions and accounts for data variance in its estimations, which requires a robust factorial model to calculate. There are several methods for assessing a scale's internal consistency. Still, the omega coefficient derived after fitting a factor analysis can be considered an acceptable demonstration of reliability for the Brazilian-Portuguese version of MA-II.

On the other hand, the multidimensional concept of HRQoL^16^ might be partially captured by the six-item MA-II^22^. One of the reasons for the low MA-II score refers to the favorable demographic characteristics of our participants: most of them were women, married, with few depressive symptoms, and currently working. In another direction, some item wordings and image cues may require adjustment, as remarked in previous studies^19,27^. Regarding the MA-II capacity to capture HRQoL, the magnitude of the ceiling effect of the total score in our study is in line with the previous estimate of 2% in the Portuguese version^19^. In other words, the ceiling effect represents a psychometric limitation when the highest possible score of a test is reached, disturbing its discriminant capacity. This finding is in contrast with the general expectation of poor HRQoL of patients with high BMI^17,20^. Therefore, further investigations should clarify how well the MA-II could capture HRQoL in different language versions, cultural settings, and surgical contexts.

Concerning physical domains, studies showed that obese patients who do not enjoy physical exercise tend to be sedentary^32^. This factor can induce weight gain, which jeopardizes the global HRQoL^17^. Several findings corroborate our results^7,17,19,20,28^, where BMI predicted poor HRQoL. Regarding work satisfaction, patients with obesity and associated medical problems tend to take off work due to health issues^15^. The correlation between BMI and capability to work can be supported through improvements in labor productivity and the functioning of patients after bariatric surgery^15^. Weight loss and the recovery of associated medical problems could improve efficiency and satisfaction with work. A sedentary lifestyle and unproductivity at work, commonly present in obese population, are also associated with depressive disorders^32^, which directly affect HRQoL. Thus, it is essential to emphasize that the MA-II was sensitive to identifying the association between the patient's BMI and the domain of physical activities and job satisfaction.

Psychiatric disorders are highly prevalent conditions among obese patients. Approximately half of the patients with low HRQoL had at least one co-occurring psychiatric diagnosis^9^. The social functioning level indicated a substantial social impairment in multiple areas^8,23^. Depressive symptoms are disabling, so it should be one of the main factors to be considered in HRQoL^9^. In the first validation study of the MA-II^22^, the total score of the questionnaire was correlated with the widely used Beck Depression Inventory-II (BDI-II). In our study, we chose the observer-based interview MADRS to rate the severity of depressive symptoms among bariatric patients^12^. In summary, the higher the total score of MA-II, the fewer depressive symptoms. Our data are in line with the literature indicating that depression and mental health can directly influence the patient's QoL^15,25^.

The comparison between standard measures of the functional dimension of HRQoL in a bariatric context is generally made through scales like the SF-36 and WHO Disability Assessment Schedule (WHODAS)^20^. Most of the assessment tools evaluate how psychopathological symptoms or medical illnesses affect the patient's day-to-day life, as patients without obesity have a good HRQoL and functionality^26^. In the present study, we used the observed-rated GAF to evaluate patients’ functionality^23,26^. While our results did not allow comparing functionality measured with SF-36 or WHODAS, our estimates indicated that the score of GAF-rated functionality was substantially associated with the MA-II.

The efficient measure of QoL involves several physical, psychological, and social aspects. Patient characteristics require adjustments to adequately explore specific domains that can reliably translate the patient's QoL. This way, the validation of the specific tools to the type of patient is required. The MA-II questionnaire was developed to evaluate the bariatric population^22,25^ and presents qualities such as playful aspects and easy-to-understand items. However, the simplicity of the questionnaire also affects its effectiveness in assessing all aspects involving HRQoL^19^. Therefore, to better evaluate this construct, the health professional should use tools with evidence of validity to understand which variables could affect the patient's perception of HRQoL.

A limitation of the present study is the participation bias. Our non-probabilistic consecutive sampling was composed of patients from a waiting list for bariatric surgery at a university-based single center. These patients were not representative of the population with severe obesity, which hampers its external generalizability. It is possible that non-bariatric obese patients could present different score ranges of HRQoL. In addition, the social desirability bias of patients displaying high expectations towards the authorization and eligibility for undergoing surgery may influence participants to self-rate themselves in a more positive light or better HRQoL.

## CONCLUSIONS

The MA-II is a valid and reliable psychometric tool to evaluate the HRQoL. This questionnaire is a helpful and quick tool for assessing the HRQoL of Brazilian patients. The existence of a psychometrically sound HRQoL tool to assess patients with psychiatric comorbidities is an asset in measuring changes in all stages of treatment. Future studies should focus on the individual variability in the performance of the tool related to sex, education, and culture of patients with severe obesity, as well as its capacity to measure HRQoL in post-bariatric and non-obese populations.
